# Critical residues involved in tau binding to fyn: implications for tau phosphorylation in Alzheimer’s disease

**DOI:** 10.1186/s40478-016-0317-4

**Published:** 2016-05-18

**Authors:** Dawn H. W. Lau, Marte Hogseth, Emma C. Phillips, Michael J. O’Neill, Amy M. Pooler, Wendy Noble, Diane P. Hanger

**Affiliations:** Department of Basic and Clinical Neuroscience, King’s College London, Maurice Wohl Clinical Neuroscience Institute (K1.24), 125 Coldharbour Lane, London, SE5 9NU UK; Eli Lilly and Company Limited, Lilly Research Centre, Erl Wood Manor, Windlesham, Surrey, GU20 6PH UK; Present address: Nestlé Institute of Health Sciences, Lausanne, Switzerland

**Keywords:** Tau, Fyn, SH3, Alzheimer’s disease, PXXP motif, Phosphorylation

## Abstract

Alzheimer’s disease (AD) is a progressive neurodegenerative disorder characterised by neuropathological deposits of amyloid plaques and neurofibrillary tangles comprised of β-amyloid and tau protein, respectively. In AD, tau becomes abnormally phosphorylated and aggregates to form intracellular deposits. However, the mechanisms by which tau exerts neurotoxicity in disease remain unclear. Recent studies have suggested that the presence of tau at synapses may indicate a role in neuronal signalling, which could be disrupted in pathological conditions. The non-receptor-associated tyrosine kinase fyn is located at the dendrite in neurons, where it was recently shown to interact with tau to stabilise receptor complexes at the post-synaptic density. Fyn also co-localises with tau in a proportion of neurons containing tau tangles in AD and fyn is also a tau kinase. Hence, tau-fyn interactions could play a pathogenic role in AD. Here we report the identification of critical proline residues, Pro213, Pro216, and Pro219, located within the fifth and sixth Pro-X-X-Pro motifs in the proline-rich region of tau, that are important for its binding to fyn. These residues in tau are flanked by numerous phosphorylation sites and therefore we investigated the relationship between fyn and the degree of tau phosphorylation in human post-mortem brain tissue. We found no difference in the amount of fyn present in control and AD brain. Notably, however, there was a significant correlation between fyn and phosphorylated tau at specific phospho-epitopes in control, but not in AD brain. Our results suggest that the pathological mechanisms underlying AD, that result in increased tau phosphorylation, may disrupt the physiological relationship between tau phosphorylation and fyn.

**Electronic supplementary material:**

The online version of this article (doi:10.1186/s40478-016-0317-4) contains supplementary material, which is available to authorized users.

## Introduction

The microtubule-associated protein tau has long been recognised as having a role in stabilising microtubules in neurons, particularly in axons. Recent evidence however, suggests that tau is also present in dendrites where it may play a role in synaptic function [1–3]. In support of this suggestion, tau has been shown to associate with synaptic proteins, such as post-synaptic density protein 95 (PSD-95) and fyn [1, 4]. In particular, a functional role has been shown for the interaction between tau and fyn, whereby tau directs fyn to the post-synaptic density to interact with N-methyl-D-aspartate (NMDA) receptors [1]. Direct binding of endogenous tau and fyn was first shown in SH-SY5Y neuroblastoma cells [5]. Subsequently, a detailed study in mouse brain demonstrated that a small proportion of tau interacts with fyn in dendrites [1]. Using a truncated form of tau lacking the microtubule-binding domain that binds to fyn but is unable to associate with microtubules, it was shown that tau sequesters fyn, thereby excluding it from dendrites and causing fyn to be translocated into the neuronal cell body [1]. Furthermore, expression of this truncated form of tau destabilised the interaction between fyn, NMDA receptors, and PSD-95 [1]. Crossing amyloid precursor protein (APP) mutant mice with transgenic mice expressing truncated tau ameliorated the premature mortality and memory deficits, and reduced the susceptibility to excitotoxic seizures observed in APP transgenic mice [1]. These data indicate that the mis-localisation of fyn caused by truncated tau rescued amyloid-induced phenotypes. Therefore, tau-fyn interactions could play a functional role in Alzheimer’s disease (AD). We have previously demonstrated a role for fyn in the dynamic targeting of tau to the plasma membrane in neurons [6]. We found that the phosphorylation status of tau affects its trafficking between the cytosol and the membrane in neurons, and that this was dependent on the region of tau that is phosphorylated [6]. Thus there is mounting evidence that tau-fyn interactions could be involved in new functionalities for tau at dendrites and in synaptic signalling [7, 8].

We focused here on identifying the binding site(s) for the tau-fyn interaction. It is well known that SH3 domains of src family kinases, of which fyn is a member, bind to PXXP motifs [9]. Tau has seven such motifs, some overlapping in tandem, within the proline-rich region in the N-terminal half. Therefore it is of interest to determine which of the motifs is critical for tau-fyn interactions. Previous studies to identify the critical PXXP motif have suggested that the sixth and seventh PXXP motifs (P216–P219 and P233–P236, respectively) are important for tau-fyn binding [10, 11], while other studies suggested that the seventh PXXP motif, rather than the sixth PXXP motif, was more important for tau-fyn binding [1, 5].

In this study, we extend the findings from Lee et al. [5] and Usardi et al. [10] to include investigation of the remaining PXXP motifs on tau using full-length, intact tau to avoid structural changes. Furthermore, since phosphorylation at serine and threonine residues affects interactions between tau and fyn [7] and the relationship between tau and fyn has not been explored extensively in humans, we investigated whether phosphorylation of tau around these motifs would correlate with levels of fyn in human post-mortem brain tissue from unaffected individuals or patients with AD.

## Materials and methods

### Plasmids

A plasmid expressing the longest isoform of human CNS tau containing two N-terminal inserts and four microtubule-binding repeats (2N4R) has been described previously [12]. The generation of plasmids encoding 2N4R tau containing proline-to-alanine substitutions at residues 216 (P216A) or 233 (P233A) of tau has been described previously [10]. Site-directed mutagenesis (QuikChange II XL, Agilent Technologies) was used to produce 2N4R tau constructs harbouring individual proline-to-alanine substitutions, P176A, P182A, P200A, P206A, P213A, and P219A. The primers used are shown in Table 1. Wild-type (WT) and mutant tau cDNA were subcloned into the pcDNA3.1-V5-His-TOPO vector (Invitrogen) to generate plasmids expressing V5-His-tagged tau. Plasmids encoding glutathione-S-transferase (GST), or GST fused to the N-terminus of the src homology (SH) two or three domains of fyn were a gift from Dr S. Anderson (University of Colorado Anschutz Medical Campus, Aurora, CO, USA) [13].

**Table 1:** Primers used for site-directed mutagenesis

Primer name	Primer sequence 5′ to 3′
Forward P176A	AGCAAAAACCGCTCCCGCTCCAAAG
Reverse P176A	CTTTGGAGCGGGAGCGGTTTTTGCT
Forward P182A	CGCTCCAAAGACAGCACCCAGCTCTGG
Reverse P182A	CCAGAGCTGGGTGCTGTCTTTGGAGCG
Forward P200A	TACAGCAGCGCAGGCTCTCCAGGCACTC
Reverse P200A	GAGTGCCTGGAGAGCCTGCGCTGCTGTA
Forward P206A	GGCTCCCCAGGCACTGCCGGCAGC
Reverse P206A	GCTGCCGGCAGTGCCTGGGGAGCC
Forward P213A	GCTCCCGCACCGCGTCCCTTCCA
Reverse P213A	TGGAAGGGACGCGGTGCGGGAGC
Forward P219A	CCCTTCCAACCCCAGCCACCCGGG
Reverse P219A	CCCGGGTGGCTGGGGTTGGAAGGG

Primers used for site-directed mutagenesis of tau. The codons mutated to replace proline (P) with alanine (A) in the encoded protein are underlined

### Cell culture and transfection

Chinese hamster ovary (CHO) cells were maintained in Ham’s F-12 (GE Healthcare) supplemented with 10 % (v/v) foetal bovine serum (Biosera), 2 mM L-glutamine, 100 U/ml penicillin, and 100 μg/ml streptomycin, and incubated in a 5 % CO_2_ atmosphere at 37 °C. Cells were plated at a density of 200,000 per well of a 6-well plate and plasmids were transfected 24 h after plating using jetPEI® (Polyplus Transfection), according to the manufacturer’s instructions. Cells were harvested 24 h after transfection.

### Pull-downs with glutathione S-transferase fusion proteins

Frozen glycerol preparations of *E.coli* containing GST- and GST-fyn-SH3-encoding plasmids were streaked across agar plates and incubated overnight at 37 °C. A single bacterial colony was used to inoculate 5 ml LB-amp broth and incubated overnight at 37 °C with shaking, before further inoculating 250 ml LB-amp broth and incubating for two hours at 37 °C with shaking until an optical density of 0.6–1.0 nm was reached. 1 mM isopropyl-1-thio-β-D-galactopyranoside (IPTG) was added to the cultures and incubated for a further 2 h with shaking. The culture was then centrifuged at 20,000 g_(av)_ for 15 min at 4 °C to pellet the bacteria. The pellet was resuspended in 12 ml TNE buffer (25 mM Tris-HCl, pH 7.5, 100 mM NaCl, 1 mM ethylenediaminetetraacetic acid (EDTA), and complete protease inhibitor cocktail without EDTA [Roche]) and homogenised by sonication (Vibra-Cell™, Sonics & Materials Inc.) for six 15 s bursts on ice. Triton X-100 was added to a final concentration of 1 % (v/v), mixed, and the samples were centrifuged at 30,000 g_(av)_ for 10 min at 4 °C. The supernatant, containing GST-fusion proteins, was collected and stored on ice. A 50 % (v/v) slurry of glutathione-4B beads (GE Healthcare, Buckinghamshire, UK) was washed three times in TNE buffer and added to supernatant containing GST-fusion proteins and rotated for 90 min at 4 °C, then centrifuged at 3300 g_(av)_ for 1 min at 4 °C to pellet the beads. Beads coupled to GST-fusion proteins were washed three times with TNE buffer and resuspended as a 50 % (v/v) slurry.

CHO cells transfected with WT or mutant tau were washed with phosphate-buffered saline, harvested into ice-cold lysis buffer (25 mM Tris-HCl, pH 7.5, 10 % (v/v) glycerol, 0.5 % (v/v) Triton X-100, 1 mM EDTA, 1 mM ethylene glycol-bis(2-aminoethylether)-*N*,*N*,*N’*,*N’*-tetraacetic acid, 150 mM NaCl, complete protease inhibitor cocktail without EDTA [Roche], and phosphatase inhibitor cocktail 2 [Sigma]), and incubated on ice for 15 min. Lysates were centrifuged at 6000 g_(av)_ for 5 min to pellet cell debris. GST-beads (50 % slurry) were washed twice in wash buffer (50 mM Tris-HCl, pH 7.5, 2 mM EDTA, 0.5 % (v/v) Triton X-100, 150 mM NaCl, complete protease inhibitor cocktail without EDTA [Roche], and phosphatase inhibitor cocktail 2 [Sigma]) and incubated with the cell lysates. The pre-cleared cell lysates were incubated overnight at 4 °C with washed GST-beads or GST-fyn-SH3-beads. Beads were pelleted at 6000 g_(av)_ for 5 min and the supernatants were discarded. Beads were washed twice with wash buffer, then Laemmli sample buffer was added and the samples heated at 100 °C for 5 min to elute bound protein. Proteins were separated on 10 % (w/v) sodium dodecyl sulphate (SDS)-polyacrylamide gels, transferred to nitrocellulose membranes and western blots were probed with antibodies to total tau, as described below. The amount of tau bound to GST-fyn-SH3 beads was quantified using an Odyssey scanner (Li-Cor Biosciences) and standardised against the total amount of tau in the corresponding cell lysate.

### Post-mortem human brain tissue

Samples of frozen temporal cortex from post-mortem human brain (15 AD and 11 controls) were obtained from the London Neurodegenerative Diseases Brain Bank at the Institute of Psychiatry, Psychology and Neuroscience, King’s College London (details in Table 2). Samples were collected and distributed in accordance with local and national research ethics committee approvals (REC reference: 08/MRE09/38+5). Alzheimer disease-related pathology was classified using diagnostic neuropathological criteria from the consortium to establish a registry for Alzheimer’s disease (CERAD) and Braak staging [14, 15]. The average ages of the AD and control cases were 87.7 ± 1.4 years and 79.2 ± 3.6 years (mean ± s.e.m), respectively. The average post-mortem delays for the AD and control brains were 33.5 ± 5.5 h and 24.2 ± 4.8 h (mean ± s.e.m), respectively. Brain tissue was thawed on ice and homogenised at 100 mg/ml in 2× Laemmli sample buffer using a mechanical homogeniser (Ultra Turrax® T8, Werke GmbH & Co., Germany). Homogenates were briefly sonicated (Vibra-Cell™, Sonics & Materials Inc.) before being centrifuged at 16,000 g_(av)_ at 4 °C for 20 min. The supernatants were collected and stored at −80 °C.

**Table 2:** Case details for control and late-stage Alzheimer’s disease post-mortem human temporal cortex

Sample number	Sex	Age (years)	Post-mortem delay (h)	Neuropathological diagnosis/Braak stage
		Mean 84 ± 2, range 55–98 years	Mean 30 ± 4, range 6–80 h	
1	F	82	43	Control
2	F	81	17	I
3	F	92	17	Control
4	M	86	6	Control
5	F	55	12	I
6	M	80	21	Control
7	F	90	50	II
8	F	87	22	Control
9	M	81	18	I
10	M	78	10	Control
11	M	59	50	Control
12	M	80	41	VI
13	F	91	29	VI
14	M	88	46	VI
15	F	92	42	VI
16	F	80	13	V
17	F	82	69	V
18	F	84	36	V/VI
19	F	90	23	V/VI
20	F	98	3.5	IV
21	M	82	80	V/VI
22	F	89	15	IV
23	F	97	12	V
24	M	89	19	V/VI
25	M	86	26	V
26	F	87	48	VI

Human post-mortem brain tissue obtained from the London Neurodegenerative Diseases Brain Bank at the Institute of Psychiatry, Psychology and Neuroscience, King’s College London. Gender, age at death, post-mortem delay, Braak stage, and/or diagnosis are included for each case

### Sodium dodecyl sulphate-polyacrylamide gel electrophoresis and western blots

Proteins were separated on 10 % (w/v) SDS-polyacrylamide gels and transferred to nitrocellulose membranes. After blocking, membranes were incubated overnight at 4 °C with primary antibodies directed against total tau (rabbit polyclonal, 1/10,000, DAKO; mouse monoclonal CP27, 1/10,000, gift from Peter Davies), GST (goat polyclonal, 1/2000 GE Healthcare), phosphorylated tau (mouse monoclonal PHF-1, 1/5000, and CP13, 1/400, both gifts from Peter Davies; rabbit polyclonal pS214, 1/500, Abcam; rabbit polyclonal pS262, 1/500, Abcam), dephosphorylated tau (mouse monoclonal Tau-1, 1/5000, Millipore), fyn (rabbit polyclonal, 1/1000, HPA023887, Sigma), β-actin (mouse monoclonal, AC15, 1/10,000, Sigma), and neuron-specific enolase (mouse monoclonal, BBS/NC/VI-H14, 1/10,000, DAKO). Blotted membranes were incubated with secondary antibodies (Alexa Fluor® 680 goat anti-mouse, 1/10,000, Life Technologies or IRDye™ 800 conjugated goat anti-rabbit, 1/10,000, Rockland Immunochemicals Inc.) and antigens were visualised and quantified using an Odyssey scanner (Li-Cor Biosciences).

### Colloidal Coomassie blue staining of polyacrylamide gels

For analysis of GST proteins bound to glutathione beads, SDS-polyacrylamide gels were incubated in ProtoBlue Safe Working Solution (National Diagnostics) and protein bands were imaged using an Odyssey scanner (Li-Cor Biosciences).

### Statistical analyses

Data were tested for normal distribution using the D’Agostino-Pearson normality test. Normally distributed samples were analysed for statistical significance (*p* < 0.05) using Student’s *t*-test or ANOVA. Data that were not normally distributed were analysed using Mann-Whitney or Kruskal-Wallis tests. Pearson’s or Spearman’s rank correlation was used to determine correlations between proteins, as appropriate. GraphPad Prism five was used for all statistical analyses.

## Results

### Interaction between the fyn-SH3 domain and tau is modulated by PXXP motifs in tau

**Fig. 1:**
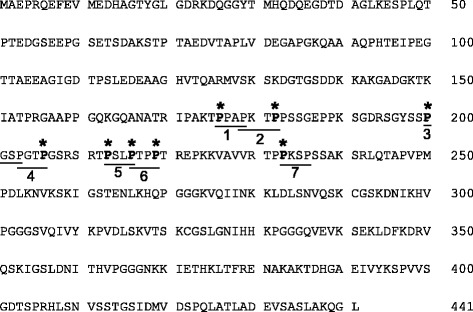
PXXP motifs in the proline-rich region of full-length human tau. The amino acid sequence of the longest isoform of human CNS tau is shown, with the PXXP motifs underlined and numbered (1–7). Individual proline to alanine substitutions within the PXXP motifs are indicated by asterisks

**Fig. 2:**
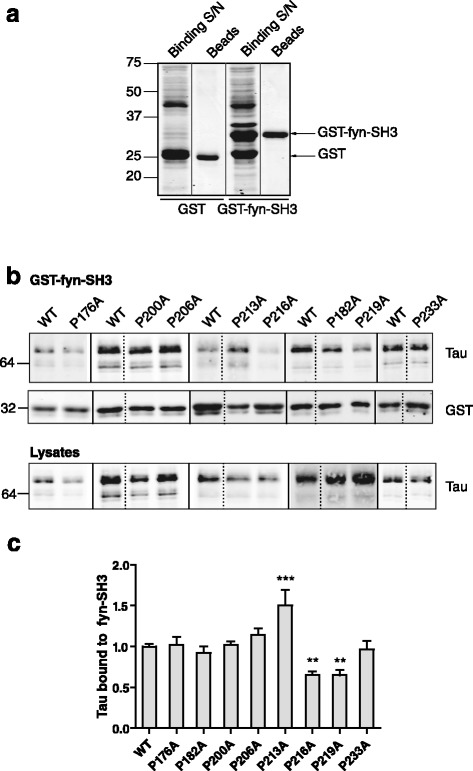
Identification of PXXP motifs critical for tau binding to fyn-SH3. **a** Coomassie blue-stained gels of supernatants from cleared bacterial lysates following induction of protein expression (Binding S/N), show glutathione *S* transferase (GST) and GST-fyn-SH3 at the expected sizes of 27 and 36 kDa, respectively. Specific coupling of GST or GST-fyn-SH3 proteins is shown (Beads). Molecular weight markers (kDa) are shown on the left. **b** Western blots of GST-fyn-SH3 pull-downs from CHO cells expressing wild-type (WT) or mutant P → A tau probed with antibodies recognising tau (upper panel) or GST (middle panel). CHO cell lysates were probed with tau antibody for standardisation (lower panel). Lanes spliced from the same blot are indicated by a dotted line. Molecular weight markers (kDa) are shown on the left. **c** Quantification of the amount of tau immunoreactivity pulled down by GST-fyn-SH3, as a proportion of WT tau, following standardisation to total tau in the corresponding lysate. Values represent mean ± SEM, *n* = 6–22. One-way ANOVA with post-hoc Dunnett’s test, **p < 0.01, ***p < 0.001

To determine which PXXP motif is important for binding of tau to fyn-SH3, we used a GST pull-down assay to test fyn-SH3 binding with WT tau and eight mutant PXXP tau constructs (P176A, P182A, P200A, P206A, P213A, P216A, P219A, and P233A) in which individual proline residues were substituted by alanine (Fig. 1). GST fusion proteins (GST-only or GST-fyn-SH3) coupled to glutathione beads were prepared and the purity of the GST proteins bound to the beads was assessed on Coomassie blue-stained polyacrylamide gels. Following induction of protein expression with IPTG, GST or GST-fyn-SH3 was readily observed in the bacterial cell lysate (Fig. 2a). Analysis of bound proteins showed single bands at the predicted sizes for GST (27 kDa) or GST-fyn-SH3 (32 kDa).

CHO cells were transfected with WT or mutant PXXP tau and cell lysates were incubated with GST or GST-fyn-SH3 beads. Proteins bound to the beads were analysed on western blots probed with an antibody against total tau (Fig. 2b). The amount of tau bound to fyn-SH3 beads was quantified and standardised against the total amount of tau in each cell lysate (Fig. 2c). Western blots of the fyn-SH3 beads showed that the amounts of P216A and P219A tau bound to fyn-SH3 was significantly decreased to ~65 % of SH3-bound WT tau (Fig. 2b, c; *p* < 0.01). Interestingly, the amount of P213A tau bound to fyn-SH3 was significantly increased by 50 % compared to WT tau (Fig. 2b, c; *p* < 0.001). There were no significant differences between the amounts of any of the other PXXP tau mutants tested (P176A, P182A, P200A, P206A, or P233A), compared to WT tau bound to fyn-SH3 (Fig. 2b, c). These results indicate that the PXXP motif at P216-P219 of tau is important for maintaining interactions between tau and fyn-SH3. Furthermore, our findings show that disruption of a proline residue closely apposed to the N-terminus of the sixth PXXP motif also increases the interaction of tau with fyn-SH3.

In agreement with previous studies reported by our group and others [10, 11], binding of tau to fyn-SH3 was unaffected by substitution of P233 with alanine in the seventh PXXP motif in tau (Fig. 2b, c). Despite reports that removal of the PXXP motif at 233–236 of tau inhibits its binding to fyn-SH3 [5], our data supports the idea that PXXP motifs N-terminal to P233 in tau are more important for tau-fyn binding. However, it should be noted that whilst both the individual P216A and P219A tau mutations reduced tau binding to fyn-SH3 by approximately 35 %, neither mutation completely abolished the interaction of tau with fyn-SH3. Thus it is likely that there are other regions of tau, in addition to the individual proline residues located inside the PXXP motifs, which are important for tau binding to fyn-SH3.

### Tau phosphorylation in Alzheimer’s disease brain is unchanged at sites surrounding critical PXXP motifs

**Fig. 3:**
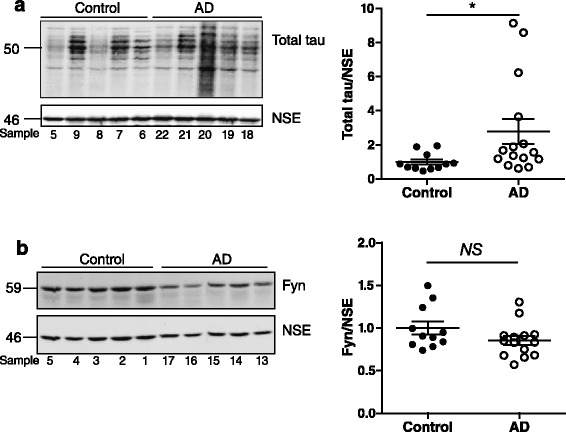
Total tau is increased in AD brain whilst the amount of fyn is unchanged. **a** Western blots of control and Alzheimer’s disease (AD) post-mortem human brain tissue probed with antibodies to total tau (upper panel) and neuron-specific enolase (NSE, lower panel). The amount of tau was quantified relative to NSE in each sample (right). **b** Western blots of control and AD brain tissue probed with antibody against fyn (upper panel). The amount of fyn was quantified relative to NSE in each sample (right). Molecular weight markers (kDa) are shown on the left. Values represent mean ± SEM, *n* = 11–15 per group. Mann-Whitney non-parametric test, **p < 0.01, *NS* = not significant

We and others have shown previously that tau phosphorylation can reduce its binding to fyn-SH3 [6, 7, 16]. The PXXP motif at residues 216–219 resides within a region of tau that is enriched in serine/threonine phosphorylation sites [17]. Thus abnormal phosphorylation of tau in AD could result in altered interactions between tau and fyn-SH3. To determine the relationship between tau phosphorylation and fyn, post-mortem human brain tissue from controls (unaffected individuals and Braak stages I–II) and from individuals with moderate to late stage AD (Braak stages IV–VI) at autopsy was analysed by western blot [18]. Probing with an antibody against tau showed that, following standardisation to neuron-specific enolase (NSE), the total amount of tau is highly variable in post-mortem human brain, particularly in AD (Fig. 3a). NSE standardisation allows for variable neuronal loss due to differing degrees of neurodegeneration in the human brain samples. The total amount of tau present in AD brain at Braak IV–VI stages was significantly increased compared to control brain (Fig. 3a; *p* < 0.01), in agreement with previous reports [19, 20]. Probing the blots with an antibody against fyn showed that after standardising to NSE, fyn was more consistent within both control and AD brain, and that the total amount of fyn was not significantly different between control and AD brain (Fig. 3b).

**Fig. 4:**
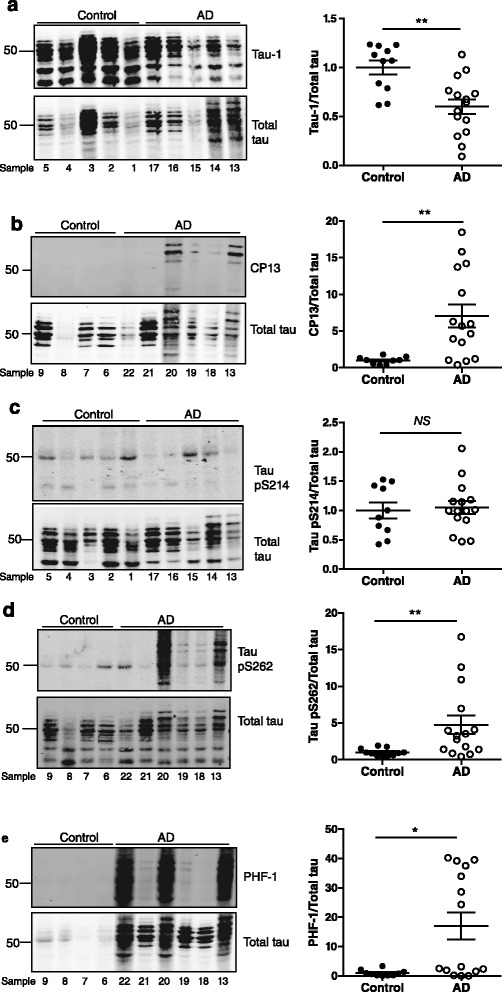
Altered tau and fyn expression at late stages of AD. Western blots of control and Alzheimer’s disease (AD) post-mortem human brain tissue probed with antibodies recognising (**a**) tau dephosphosphorylated at S199/S202 (Tau-1), **b** tau phosphorylated at S202 (CP13), **c** tau phosphorylated at S214, **d** tau phosphorylated at S262, and (**e**) tau phosphorylated at S306/S404 (PHF-1). Quantification of each epitope relative to total tau is shown on the right. Molecular weight markers (kDa) are shown on the left. Values represent mean ± SEM, *n* = 11–15 per group. Mann-Whitney non-parametric test, *p < 0.05, **p < 0.01, *NS* = not significant

Next, we analysed the human post-mortem brain tissue for tau phosphorylation, particularly at serine/threonine residues lying in close proximity to the proline-rich region, and also towards the C-terminus of tau. Tau phosphorylation was standardised to total tau, to take into account the wide variation in the amount of tau present in each brain sample. Tau-1, an antibody which recognises tau when it is dephosphorylated at S199/S202, revealed prominent bands corresponding to normally phosphorylated tau in all samples tested (Fig. 4a). Quantification of Tau-1 showed a 40 % decrease in Tau-1 immunoreactivity in AD compared to control brain, indicating increased phosphorylation of S199/S202 tau in AD, as expected [21] (Fig. 4a; *p* < 0.01). Tau immunoreactivity to CP13 antibody (tau phosphorylated at S202) was barely detectable in controls but immunoreactive bands at ~65–70 kDa were recognised by this antibody in AD brain (Fig. 4b), as shown previously [22]. The Odyssey imaging system has a dynamic range of several orders of magnitude and is capable of detecting immunoreactive bands that may not be visible by eye, as demonstrated by the presence of CP13-immunoreactive bands in control brain tissue in high-intensity scans (Additional file 1: Figure S1a). Quantification of these CP13-immunoreactive tau species relative to total tau revealed that phosphorylation at S202 of tau is significantly increased in AD brain compared to controls (Fig. 4b; *p* < 0.01). Quantification of blots probed for phosphorylation of tau at S214, located within the fifth PXXP motif, showed that this site on tau is phosphorylated similarly in both AD and control brain, although the degree of phosphorylation showed a larger degree of variation in control brain (Fig. 4c). Similarly to CP13, the pS262 tau antibody detected 65–70 kDa species in AD brain but not in controls and these tau species were also found to be significantly increased in AD, as previously shown [23] (Fig. 4d; *p* < 0.01). The PHF-1 antibody (tau phosphorylated at S396/S404) showed barely any immunoreactivity in control brain and a significant increase in AD brains compared to controls (Fig. 4e; *p* < 0.05), although weak PHF-1 immunoreactivity can be observed in control brain tissue in high intensity blots (Additional file 1: Figure S1b). Taken together, these data show that consistent with previous reports, increased phosphorylation at tau epitopes corresponding to S199, S202, S262, and S396/S404 are a feature of AD brain [20, 24–29].

### Fyn is not correlated with tau phosphorylation in Alzheimer’s disease brain

To determine whether there is a correlation between the amount of fyn protein in AD and tau phosphorylation at the epitopes investigated in this study, we performed a non-parametric Spearman, or parametric Pearson correlation analysis, depending on the normality of the data distribution. Correlation between tau and fyn was first examined in the combined cohort of control and AD patients, to investigate whether there was any association irrespective of neuropathological status. We found no significant correlation between the amount of fyn and either total tau, or tau phosphorylated at S199/S202, S202, S214, S262, or S396/S404 in the combined samples (data not shown).

**Fig. 5:**
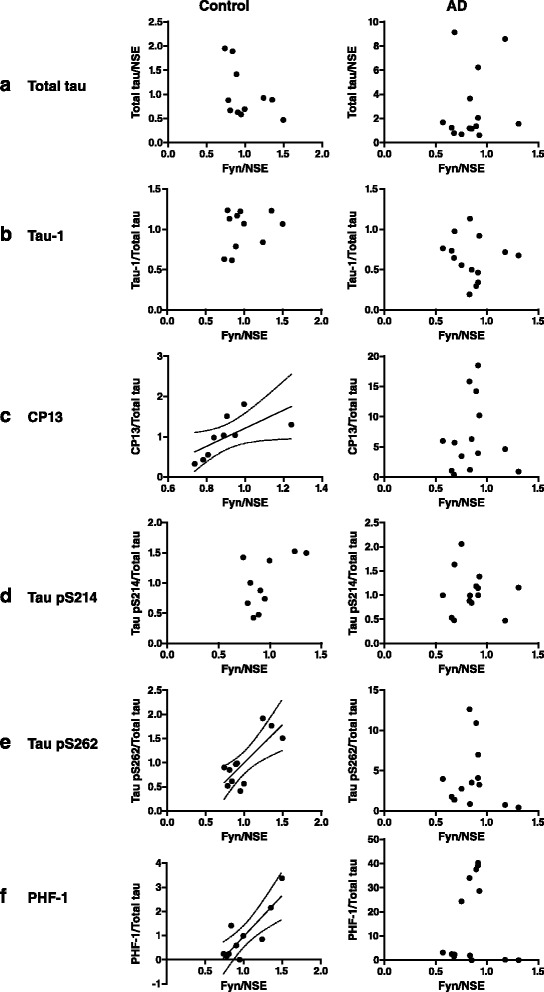
Correlation analysis of disease-related changes in tau and fyn in Alzheimer’s disease brain. **a** Total tau, expressed relative to neuron-specific enolase (NSE), and (**b**-**f**) tau phospho-epitopes Tau-1; CP13; pS214; pS262; and PHF-1, expressed relative to total tau were compared to the amount of fyn relative to NSE in post-mortem control and Alzheimer’s disease (AD) human brain. Pearson’s or Spearman’s rank correlation analysis showed significant correlation of fyn with CP13, pS262, and PHF-1 in control brain, but not in AD brain (details in Table 3), as indicated by a line of best fit

We next determined whether there was a correlation between fyn and tau in the separate control and AD cohorts that may be due to their differential neuropathological AD status (Fig. 5). A summary of the correlation coefficients and significance levels obtained for correlation between fyn and tau in control and AD brain is shown in Table 3. This analysis revealed the interesting findings that the amount of fyn present in control human brain correlated positively with the amount of tau phosphorylated at S202 (CP13), S262, and S396/404 (PHF-1), but not at S214 (Table 3). In contrast there was no correlation between fyn and phosphorylated tau in AD brain, suggesting a potential disruption in the fyn-tau relationship in AD. Our data support the hypothesis that there may be an association between tau and fyn under physiological conditions, but that pathological activation of fyn or other disease-associated alterations in fyn in AD may directly or indirectly impact regulation of tau phosphorylation, or vice versa.

**Table 3:** Correlation analysis between tau and fyn in control and Alzheimer’s disease brain

	Control	AD
Fyn vs	*r*	*r*
Total tau	−0.468	0.156
Tau-1	0.258	−0.120
CP13	0.673*	0.041
Tau pS214	0.570	−0.078
Tau pS262	0.777**	−0.121
PHF-1	0.837**	0.002

Pearson’s or Spearman’s rank correlation coefficients (*r*) corresponding to associations between fyn, and tau (total or phosphorylated). Correlation analyses were carried out on brains from either controls or Alzheimer’s disease (AD); *p < 0.05, **p < 0.01

## Discussion

The interaction between tau and fyn has recently been proposed as a possible therapeutic target for AD [11, 30–32]. Aberrant mislocalisation, or post-translational modification, of tau and/or fyn under pathological conditions could lead to altered binding between these two proteins [33]. A growing body of evidence supports the hypothesis that tau-fyn interactions play a role in regulating synaptic function [1, 8, 34]. Altered binding between tau and fyn could lead to unwanted downstream consequences in AD, triggering further cascades that lead to synaptotoxicity. In particular, tau and fyn appear to work in concert to promote NMDA receptor stability [1, 34]. Furthermore, inhibiting fyn kinase activity reduces tau aggregation in a transgenic mouse model of AD, suggesting that tau-fyn interaction could exacerbate tau pathology in AD [30, 35]. Therefore, we sought to identify the physiologically important binding site between tau and fyn, and to determine whether disease-related phosphorylation of tau was related to levels of fyn in pathogenic conditions.

In this study, we substituted individual proline residues with alanine in each of the seven PXXP motifs in tau to determine the effect on its ability to bind to the SH3 domain of fyn. We found that substitution of either P216 or P219 in tau reduces tau binding, whereas the P213A mutation increases the association of tau with fyn-SH3. This indicates that fyn-SH3 binds to the sixth PXXP motif of tau (residues 216–219), and that the fifth PXXP motif of tau at 213–216 also plays an important role in regulating tau-fyn-SH3 interaction.

We have demonstrated previously that replacing P216, but not P233, with alanine reduces tau binding to fyn-SH3 in CHO cells [10], and this finding has since been confirmed in a more recent study using bioluminescence resonance energy transfer in cells [11]. In contrast, earlier *in vitro* studies, using truncated tau constructs, have suggested that the seventh PXXP motif at residues 233–236, rather than the sixth motif (216–219) is more important for tau-fyn binding [1, 5]. The reason for this discrepancy is not known, but one possible explanation could be differential binding and conformation of full-length, intact human tau that was used in this study and others that identified the sixth motif (P216–P219) [10, 11]. In comparison, truncated tau was used in other studies that identified the seventh PXXP motif (P233–P236) [1, 5].

PXXP-containing ligands are categorised as either class I or II, with consensus sequences of RPL[PPXP] or XXX[PXXP]XR respectively, depending on whether the flanking by positively charged residues are located N or C terminal to the PXXP motif [36]. Class I and II ligands differ in the orientation in which they bind to SH3 domains and, whereas SH3 domains can exhibit a preference for either class of ligand, fyn-SH3 binds to both classes [36–38]. In contrast to previous reports in which the tau PXXP motif at 233–236 appeared to be important for binding to fyn-SH3 [1, 5], we show here that tau residues 213–219 are responsible for this interaction, with the sixth motif (P216–P219) enabling binding. Furthermore, we have identified a potential regulatory role for P213 in the fifth PXXP motif in tau since mutation of this residue results in increased binding to fyn. The amino acid sequences flanking the fifth, sixth and seventh PXXP motifs in tau are 208-SRSRT[PSLP]TPPT-220, 211-RTPSL[PTPP]TREP-223, and 228-VVRTP[PKSP]SSAK-240, respectively. Therefore, the fifth (P213–P216) and seventh (P233–P236) PXXP motifs show some similarity with class I ligands, whereas the sixth motif (P216–P219) meets the consensus requirements of a class II ligand. Although fyn-SH3 can bind to both class I and II ligands, interaction with tau could be dependent on its conformation, such that fyn-SH3 might favour binding to the sixth PXXP motif as a class II ligand. However, this has not yet been examined and the functional implications of SH3 domain binding to class I or II PXXP ligands are not well defined.

The sixth tau PXXP motif (P216–P219) appears to be the preferred binding domain for fyn-SH3, although disruption to this region of tau could enable fyn to bind to alternative PXXP motifs in tau. The fact that none of the individual proline to alanine substitutions in tau examined here completely abolished fyn-SH3 binding, suggests that fyn-SH3 has the capacity to bind to different PXXP motifs under different physiological and pathological conditions. Further support for this hypothesis comes from a study by Ittner et al. [1] who demonstrated that a truncated tau construct (residues 1–196), containing the N-terminus and the first two PXXP motifs, did not bind fyn in transfected HEK293T cells, whereas another truncated N-terminal tau construct (1–221), that included the sixth PXXP motif, exhibited reduced binding to fyn compared to full-length tau. Thus, exclusion of the seventh PXXP motif (residues P233–P236) and the remaining C-terminal residues was insufficient to completely abolish tau-fyn-SH3 binding [1], suggesting a role for other regions of tau in mediating binding with fyn-SH3.

Interestingly, we found that the P213A tau mutation resulted in an increased amount of tau bound to fyn-SH3. One possibility is that the alanine substitution at this residue alters the conformation of tau to make it more favourable for binding to fyn-SH3, providing that the sixth PXXP motif is also present. It has been shown that specific residues outside the core PXXP ligand can contribute to the binding between PXXP motifs and SH3 domains [39], which could explain the increased binding affinity between P213A mutant tau and fyn-SH3. For example, a serine residue located close to the PXXP motif is important for binding to the SH3 domain of the adaptor protein, Nck (non-catalytic region of tyrosine kinase adaptor protein 1) [40]. Residues located C-terminal to PXXP motifs can also modulate binding to fyn-SH3, although residues N-terminal to PXXP have not yet been examined [41]. Thus, if fyn-SH3 binds to tau at residues 216–219, the proline to alanine substitution at 213 of tau could result in increased binding affinity with fyn-SH3. Another possibility is that the P213A mutation may alter tau phosphorylation around this PXXP motif, which could enhance tau-fyn binding at the SH3 domain.

In a related study, we showed previously that the phosphorylation status of residues S199/S202 in tau influences its binding to fyn-SH3 [6], but the effects of tau phosphorylation at other residues on tau-fyn binding have not been determined. The proline-rich region of tau, which contains the PXXP motifs, also includes a high density of serine/threonine residues [17]. Therefore, it is likely that phosphorylation, or other post-translational modifications, of residues surrounding the sixth PXXP motif of tau, could influence its binding to fyn-SH3. Substitution of 18 serine and threonine residues in the N-terminal half of tau to glutamate, to mimic a state of permanent phosphorylation, inhibits its binding to fyn-SH3 [7]. These mutations include 11 residues which are located within the proline-rich region of tau. Binding of tau to fyn-SH3 was also abolished using Tau-Glu10, a construct that mimics permanent phosphorylation at ten serine/threonine sites, including five in the proline-rich region [7]. Additionally, fyn-SH3 is unable to bind to highly phosphorylated tau extracted from AD brain [7]. Therefore, it appears that serine/threonine phosphorylation of tau influences its interaction with fyn-SH3, and much of this phosphorylation occurs around the PXXP motifs (P213-P219) identified here as being important for binding between tau and fyn-SH3.

However, the relationship of fyn with total tau or tau phosphorylation has not been previously investigated in AD. In agreement with previous reports, and despite the wide variation between individuals with AD, we detected increased tau in AD brain [19, 20].

We found no significant correlation between the amount of fyn present and the degree of tau phosphorylation in AD brain tissue, suggesting that modification of tau phosphorylation, at least at the serine/threonine residues examined in this study, does not result or lead to increased fyn in AD. Interestingly, in control brain tissue, although the amount of fyn did not correlate with tau phosphorylation at residues S199 or S214, there was a significant positive correlation between fyn and tau phosphorylated at S202, S262, and S396/404. The absence of this correlation in AD brain suggests that, at least during moderate to late stages of AD (Braak IV–VI), underlying pathogenic mechanisms may disrupt the association between fyn and tau phosphorylated at S202, S262, and S396/404. Notably, inhibition of casein kinase 1 delta, which phosphorylates tau [42], in primary cortical neurons, results in dephosphorylation of tau at multiple sites, including S202, and increases the association of tau with fyn-SH3 [6]. In contrast, increasing tau phosphorylation using the protein phosphatase inhibitor okadaic acid decreases tau binding to fyn-SH3 [6]. An aspartate phosphomimic of tau at S202 has altered affinity for fyn *in vitro*, dependent on the presence of the microtubule-binding repeats of tau [16]. Phosphorylation at S262 of tau has been shown to result in detachment of tau from microtubules and decreased microtubule stability, as well as inhibition of tau aggregation [42, 43]. Our results could indicate therefore that disassociation of tau from microtubules might affect fyn expression, or that the presence of fyn could influence tau phosphorylation at these residues when tau is not bound to microtubules. It is plausible that tau phosphorylated at S202 and S262 can bind to fyn or influence fyn expression or function under basal conditions, but at late stages of AD, tau becomes increasingly phosphorylated at other residues which could alter the its subcellular localisation or conformation and cause the loss of tau-fyn binding. The progressive accumulation of tau into aggregates in AD could also result in the loss of the association with fyn. Additionally, it was recently shown that tau phosphorylation at S396 is induced following NMDA-receptor mediated long-term depression [4]. Indeed, NMDA receptor activation increased the association of tau phosphorylated at S396/404 with fyn [4]. Taken together, the significant correlation of phosphorylated S396/404 tau with fyn in control brain tissue supports the idea that NMDA receptor activation enhances tau-fyn association. Conversely, the lack of significant correlation between tau phosphorylated at this epitope and fyn in AD brain suggests that pathogenic disease-related mechanisms could interfere with the relationship between phosphorylated tau and fyn. Additional studies involving analysis of fyn kinase activity would likely provide more insight as to the role of fyn in regulating downstream effects of tau phosphorylation and vice versa. Hence, additional investigation is required to fully understand the relationship between fyn and tau phosphorylation, in particular at residues S202, S262, and S396/404 in AD brain.

## Conclusions

In this study, identification of the binding site between tau and fyn-SH3 may facilitate the development of compounds that can inhibit tau-fyn interactions, which presents an alternative therapeutic strategy for AD. We also provide evidence that a physiological correlation between phosphorylated tau at S202, S262, and S396/404 and fyn is not present in AD brain, suggesting that progression of disease pathogenesis can influence the relationship between tau and fyn. Further investigation as to the relationship between fyn kinase activity and tau phosphorylation will be important in developing novel therapeutics for AD.

## Additional file

Additional file 1: Figure S1. High intensity scans of western blots of control and Alzheimer’s disease (AD) post-mortem human brain tissue from Fig. 4 reveal weak detection of tau phosphorylated at (a) S202 (CP13) and (b) S396/S404 (PHF-1) in control brain tissue, whereas the signal in AD brain is overexposed when shown at this intensity. The molecular weight marker (50 kDa) is shown on the left. (PDF 2638 kb)

## Abbreviations

AD: Alzheimer’s disease
APP: amyloid precursor protein
CHO: Chinese hamster ovary
CNS: central nervous system
EDTA: ethylenediaminetetraacetic acid
GST: glutathione-S-transferase
IPTG: isopropyl-1-thio-β-D-galactopyranoside
NMDA: N-methyl-D-aspartate
NSE: neuron-specific enolase
PSD-95: post-synaptic density protein 95
SDS: sodium dodecyl sulphate
SH: src homology
WT: wild-type
