# Supplementary figures and images for: Critical residues involved in tau binding to fyn: implications for tau phosphorylation in Alzheimer’s disease
Source: Acta Neuropathol Commun. 2016 May 18;4:49. doi: 10.1186/s40478-016-0317-4 (PMC4870772; doi:10.1186/s40478-016-0317-4)

**a**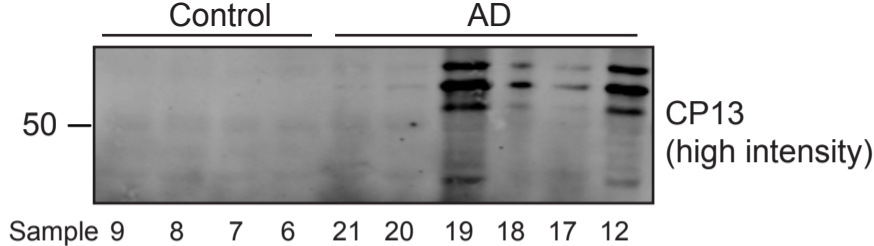**b**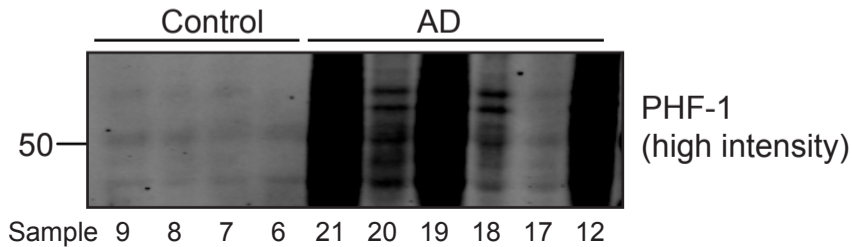

Supplement: Additional file 1: Figure S1. — High intensity scans of western blots of control and Alzheimer’s disease (AD) post-mortem human brain tissue from Fig. 4 reveal weak detection of tau phosphorylated at (a) S202 (CP13) and (b) S396/S404 (PHF-1) in control brain tissue, whereas the signal in AD brain is overexposed when shown at this intensity. The molecular weight marker (50 kDa) is shown on the left. (PDF 2638 kb) [file 40478_2016_317_MOESM1_ESM.pdf]
